# Supplementary material for: Lack of Cathepsin D in the Renal Proximal Tubular Cells Resulted in Increased Sensitivity against Renal Ischemia/Reperfusion Injury
Source: Int J Mol Sci. 2019 Apr 5;20(7):1711. doi: 10.3390/ijms20071711 (PMC6479628; doi:10.3390/ijms20071711)
Supplement: Supplementary file 1 [file ijms-20-01711-s001.pdf]

Supplementary Materials

Lack of Cathepsin D in the Renal Proximal Tubular Cells Resulted in Increased Sensitivity Against Renal Ischemia/Reperfusion Injury

Chigure Suzuki, Isei Tanida, Masaki Ohmuraya, Juan Alejandro Oliva Trejo, Soichiro Kakuta, Takehiko Sunabori and Yasuo Uchiyama

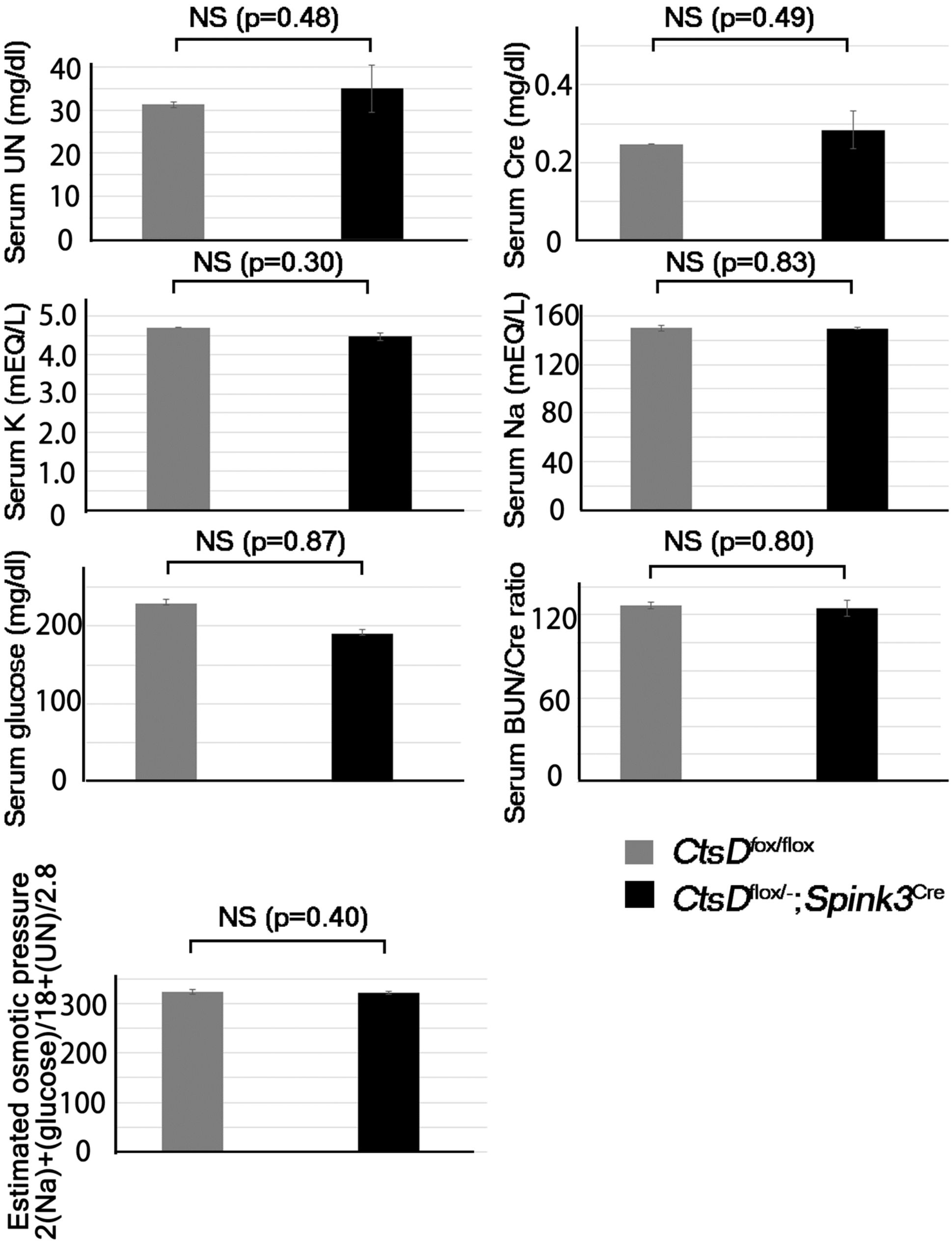

Figure S1. Negligible difference in blood tests between *CtsD*$^{flox/-}$; *Spink3*^{Cre} and Control mice**.** Blood tests of *CtsD*$^{flox/-}$; *Spink3*^{Cre} and control mice were performed at 48 hours after ischemia/reperfusion (I/R) injury. We performed hemi-nephrectomy ahead of I/R because unilateral I/R injury barely induced clear abnormality and bilateral I/R resulted in a large dispersion of data owing to individual differences in wild type mice. Data in graphs are expressed as the mean ± SEM. Statistical analyses

were performed using a student's t-test, and statistical significance was set at p<0.05. **NS**: no significant difference, **UN**: urea nitrogen, **Cre**: creatinine, **K**: potassium, **Na**: sodium. *n* = 3 to 4.

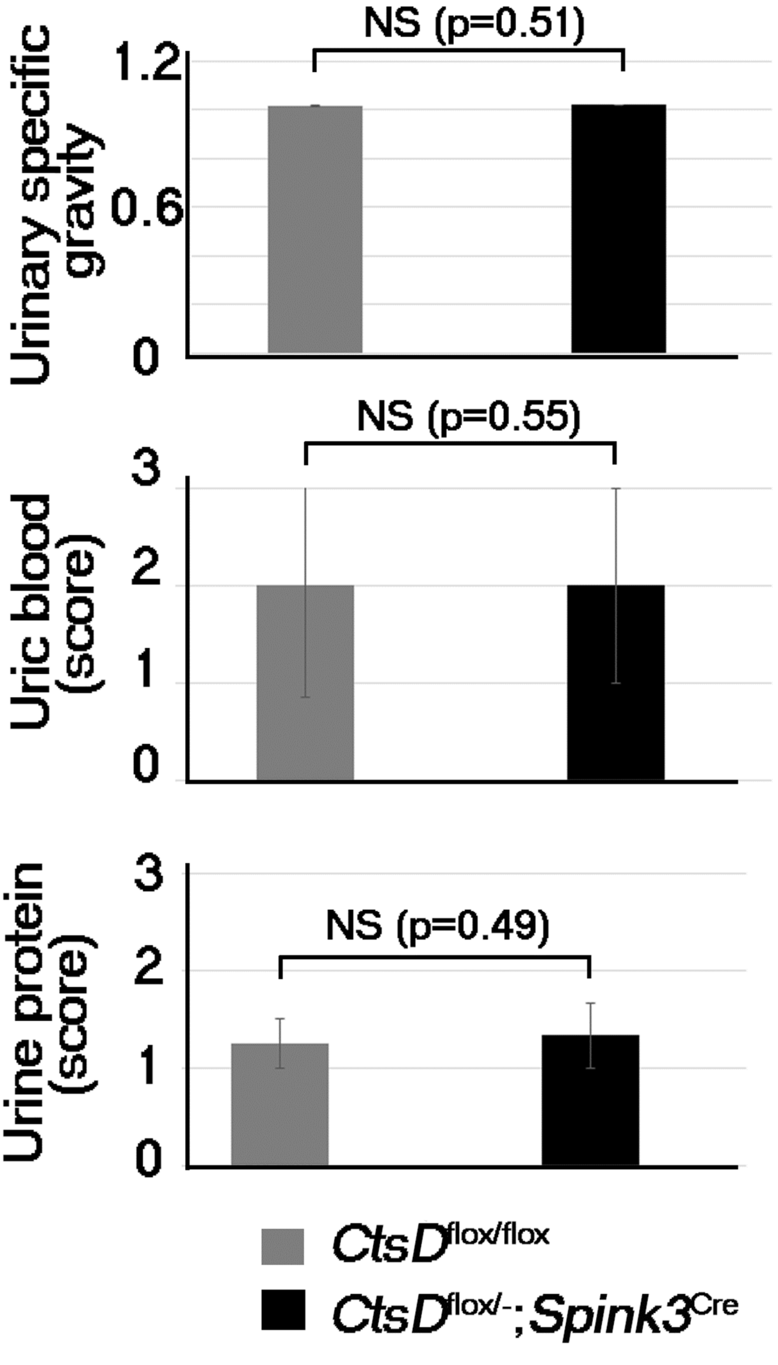

Figure S2. Negligible difference in urinalysis between *CtsD*$^{flox/-}$; *Spink3*^{Cre} and Control mice. Urine analyses were performed in *CtsD*$^{flox/-}$; *Spink3*^{Cre} and control mice 48 hours after ischemia/reperfusion (I/R) injury with hemi-nephrectomy. **Urinary specific gravity**, **Ureic blood** and **Urine protein** are shown (n = 3). Data in graphs are expressed as the mean ± SEM. Statistical analyese were performed using a student's t-test, and statistical significance was set at p<0.05.

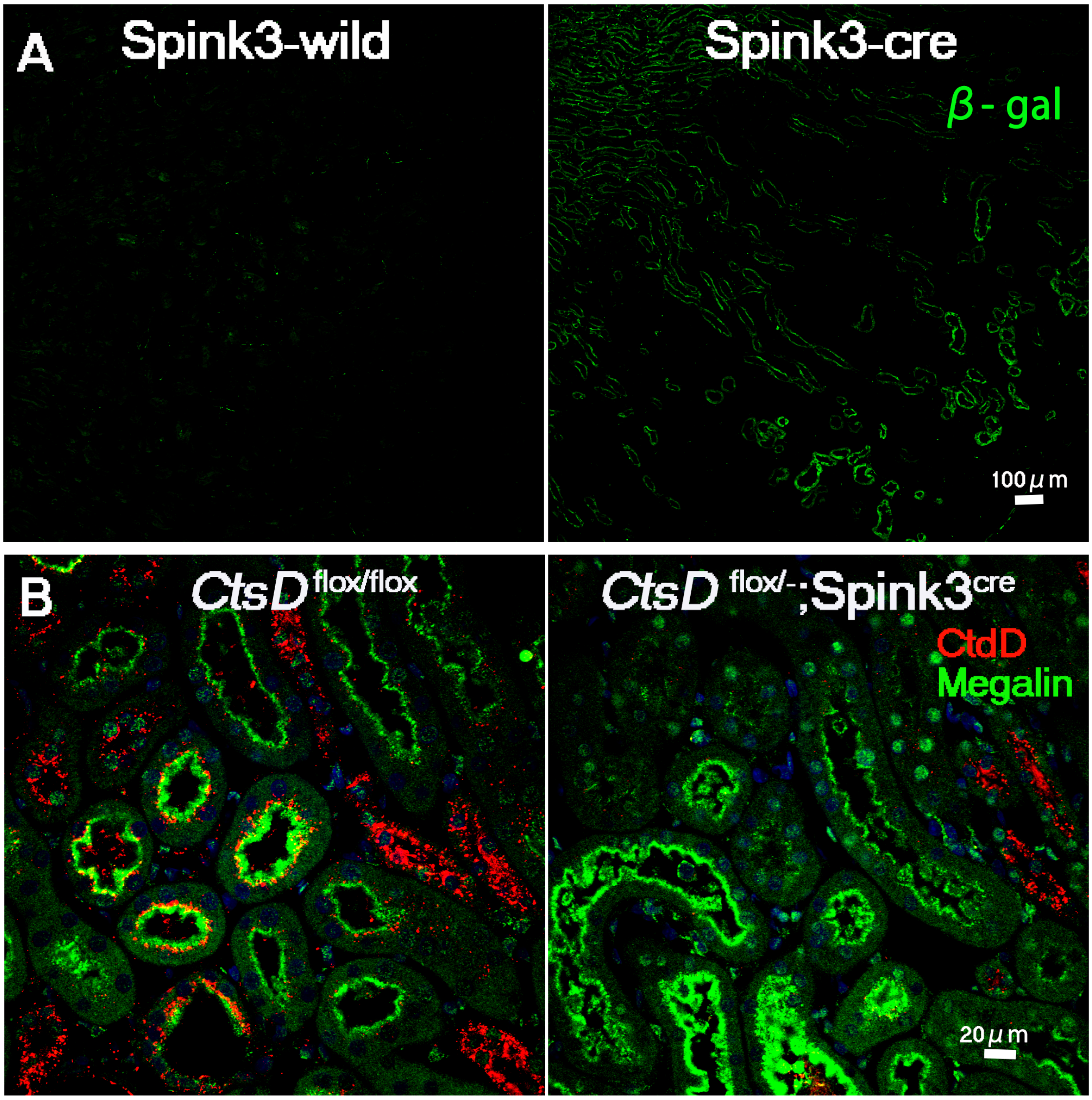

Figure S3. Megalin, a marker of renal proximal tubular cells, positive cells lacked cathepsin D-immunopositive signals in *CtsD*$^{flox/-}$; *Spink3*^{Cre} mice. (**A**) X-gal staining of *Spink*3-cre mouse kidneys for confirmation of *Spink*3 expression in renal tubular cells (**Green**). Tubular cells in the cortico-medullary region were stained. (**B**) Double staining of cathepsin D (**Red**) and Megalin (a marker of proximal tubular cells, **Green**). No **red** signals of cathepsin D were detected in the Megalin positive (**Green**) proximal tubular cells of *CtsD*$^{flox/-}$; *Spink3*^{Cre} mice.

Supplemenntary Methods

X-gal staining

To determine *Spink*3 promoter activity, we detected the activity of βgalactosidase produced by the knock-in lacZ. *Spink*3-Cre mice were bred with Rosa26R-Z mice. ROSA26 is a locus used for constitutive, ubiquitous gene expression in mice [1].

At 6 weeks of age, these mice were anesthetized and fixed by intracardial perfusion. Kidneys were removed and embedded in O.C.T compound and frozen. X-gal staining was done as described previously [2]. In brief, 8 μm frozen sections of *Spink*3-wild; R26R and *Spink*3-cre; R26R mice were

blocked with 0.1% BSA for 1 hour and incubated with rabbit anti-β galactosidase antibody (cappel, CA, USA) in 1% BSA overnight. After washing anti-rabbit Alexa488 and DAPI were added and incubated at room temperature for 1 hour. Images were taken using a Fluorescence microscope FV1000 (OLYMPUS).

Biochemical analysis

Mice were anesthetized and blood was withdrawn by cardiac puncture. Blood samples were centrifuged for 15 min at 3,000 g at room temperature and sera were collected. The sera were biochemically analyzed via Oriental Yast Co,ltd. Urine samples were collected before collecting blood and tested using urine test paper (Uro-Hema-Combistix SG-L; Siemens Healthcare Diagnostics).

References

1. Soriano, P., Generalized lacZ expression with the ROSA26 Cre reporter strain. *Nat Genet* **1999,** *21*, (1), 70–71.
2. Wang, J.; Ohmuraya, M.; Hirota, M.; Baba, H.; Zhao, G.; Takeya, M.; Araki, K.; Yamamura, K., Expression pattern of serine protease inhibitor kazal type 3 (Spink3) during mouse embryonic development. *Histochem. Cell Biol.* **2008,** *130*, (2), 387–397.
